# Long-range correlations and patterns of recurrence in children and adults' attention to hierarchical displays

**DOI:** 10.3389/fphys.2015.00138

**Published:** 2015-05-06

**Authors:** Ramon D. Castillo, Heidi Kloos, John G. Holden, Michael J. Richardson

**Affiliations:** ^1^Facultad de Psicología, Universidad de TalcaTalca, Chile; ^2^Department of Psychology, CAP Center for Cognition, Action, and Perception, University of CincinnatiCincinnati, OH, USA

**Keywords:** recurrence quantification analysis, detrended fluctuation analysis, 1/f noise, self-organization, local/global visual processing, dynamic systems

## Abstract

In order to make sense of a scene, a person must pay attention to several levels of nested order, ranging from the most differentiated details of the display to the integrated whole. In adults, research shows that the processes of integration and differentiation have the signature of self-organization. Does the same hold for children? The current study addresses this question with children between 6 and 9 years of age, using two tasks that require attention to hierarchical displays. A group of adults were tested as well, for control purposes. To get at the question of self-organization, reaction times were submitted to a detrended fluctuation analysis and a recurrence quantification analysis. H exponents show a long-range correlations (1/*f* noise), and recurrence measures (percent determinism, maximum line, entropy, and trend), show a deterministic structure of variability being characteristic of self-organizing systems. Findings are discussed in terms of organism-environment coupling that gives rise to fluid attention to hierarchical displays.

## Introduction

A look out the window reveals coherence at all levels of order, from the small detail of a wall, to the broad impression of a busy intersection. How are these different levels of order integrated into a coherent whole? Numerous studies have looked into this question of *local/global processing*, ranging back to the historical routes of the field (e.g., James, 1890; Köhler, 1929/1947; Koffka, 1935; Treisman, 1964). However, the question about coherent perception of hierarchical displays has not been resolved, despite the many different tasks that have been developed, administered to many different age groups (including infants and the elderly), and measuring multiple outcomes in different populations of typical and atypical development (e.g., Navon, 1977; Martin, 1979; Enns and Girgus, 1985; Kimchi, 1990, 1998, 2009; Ben-Av and Sagi, 1995; Rensink and Enns, 1995; Heinze et al., 1998; Han et al., 1999; Dukette and Stiles, 2001; Behrmann and Kimchi, 2003; Kimchi and Razpurker-Apfeld, 2004; Kimchi et al., 2005; Poirel et al., 2008; Förster, 2012).

The problem, we believe, is rooted in the intuition that local/global processing is carried out by separate processes, those that focus on integrating elements into a global whole, and those that focus on differentiating a global whole into separate elements. Despite evidence in support of this intuition, we argue instead that local/global processing is the result of a soft-assembled system that seeks an adaptive balance of constraints at all levels of order.

### Separate processes of integration and differentiation?

Intuitive support for separated processes in local/global perception comes from our phenomenological experience: We can easily focus our attention on the larger whole, and we can focus our attention on a detail. In line with this intuition, there is neurological evidence of two different pathways related to hemispheric specialization (Fink et al., 1996, 1997; Heinze et al., 1998; Beaucousin et al., 2011). Then there is the so-called global precedence effect, the finding that people attend to the global information first, giving further credence to the idea that integration and differentiation are separate processes (cf., Navon, 2003; Poirel et al., 2008; Förster, 2012). This effect was found with children, adults, and elderly; it was found with familiar and unfamiliar stimuli; and it was found with various tasks and outcome measures (e.g., Navon, 1977; Enns and Girgus, 1985; Ben-Av and Sagi, 1995; Rensink and Enns, 1995; Kimchi, 1998; Han et al., 1999; Dukette and Stiles, 2001; Behrmann and Kimchi, 2003; Kimchi and Razpurker-Apfeld, 2004).

There is a caveat however: The empirical global-precedence evidence is far messier than an automatic deployment of an integration process would imply. At the minimum, the global precedence is tied to the specific details of the stimuli: Grouping many small elements into a global configuration appears to differ from grouping a few large elements; vice versa, differentiating among few elements appears to differ from differentiating among many elements (Kimchi et al., 2005; Poirel et al., 2006, 2008). This effect of stimuli characteristics implies that a dual-process view of local/global perception is insufficient to capture the full data. It would have to be expanded to include at least four separate processes: integration of many elements, integration of few elements, differentiation of few elements, and differentiation of many elements.

In the current paper we reject the idea that local/global perception can be captured by separable processes. This is because a separate-process model would fall short of explaining how the proposed processes are coordinated to give rise to smooth visual exploration. The idea is instead that perceptual organization involves the coming together of interdependent processes that operate on different time scales, including processes in neurophysiology, motor behavior, attention, and intention. For perception to take place, they all combine into a coordinated whole of coupled processes. Similar ideas have been put forward for a variety of motor, perceptual, and cognitive behavior (for reviews, see Kloos and Van Orden, 2010; West, 2010; West and Grigolini, 2010). In fact, there is a consensus that human activity requires coordination across a multitude of neurophysiological, perceptual, and motor sub-systems that are operating at different time scales (Newell et al., 2001; Riley and Turvey, 2002; Van Orden et al., 2003; Turvey, 2007). This coordinative dynamic is the signature of adaptive functioning of human activity (Holden, 2005; Van Orden et al., 2011).

Initial evidence for the idea of coupling across scales in local/global processing comes from Castillo et al. (2015): Adults had to decide whether hierarchical compound items matched in a local element, in their global shape, or not at all. This task, to decide whether there is a match on any scale of order, is likely to tap into the same processes of local/global perception that or used during every-day explorations. Reaction time measured across a large number of trials was subjected to a spectral analysis. The fractal exponents we obtained provided evidence for non-random coupling of multiple scales, mimicking the findings for motor tasks, perceptual, tasks, and simple decision tasks (e.g., Gilden et al., 1995; Clayton and Frey, 1997; Gilden, 2001; Newell et al., 2001; Aks et al., 2002; Riley and Turvey, 2002; Ward, 2002; Aks and Sprott, 2003; Holden et al., 2011; Van Orden et al., 2003; Kello et al., 2007; Shockley et al., 2007; Turvey, 2007; Fernandes and Chau, 2008; McIlhagga, 2008; Stephen and Mirman, 2010; Kuznetsov and Wallot, 2011; Athreya et al., 2012; Coey et al., 2012; van Rooij et al., 2013; Malone et al., 2014).

Here we seek further evidence for the presence of multi-scale coupling in local/global processing, focusing explicitly on the question of development. Do the dynamics of local/global processing change with development? Existing research presents a developmental story that is far from clear. For example, while some studies find a decrease in global precedence over time (e.g., Freeseman et al., 1993; Frick et al., 2000; Cassia and Simion, 2002; Huizinga et al., 2010), others show an increase (Dukette and Stiles, 2001; Poirel et al., 2008; Scherf et al., 2009). And while there is the occasional suggestion that early local/global processing requires the coordination of multiple processes (Dukette and Stiles, 2001; Kimchi et al., 2005), an explicit test of such coordination is missing. In this paper, we seek to fill this gap, using the Detrended Fluctuation Analysis (DFA) and the Recurrence Quantification Analysis (RQA). These analyses have been used with children to estimate the degree of coupling among underlying processes relevant to walking (Hausdorff, 2007), tapping at a memorized rhythm (Kiefer et al., 2014), reading (Wallot et al., 2014), and interacting with a caregiver (Dale and Spivey, 2006; Warlaumont et al., 2010). The current study builds upon these efforts, assessing the degree of coupling among processes that give rise to local/global perception.

In brief, DFA (Peng et al., 1995) provides an index of self-similarity in the time series of response times (Bassingthwaighte et al., 1994). DFA first partitions a trial series into different size subsets. For each subset of a certain size, the best fitting trend lines are found and their root mean square residual (*Q*) is calculated. The log10 of this variation (*Q*) is plotted against log10 of the subset size. The slope of the regression line of the log10-log10 plot represents the Hurst exponent (*H*). The exponent reflects the degree of long-range correlations across the different time scales. *H* = 0.50 is indicative of randomness (white noise). This value depicts a lack of coupling of trials. By contrast, *H* > 0.50 indicates long-range correlations among trials, implying that processes operate in a connected manner as a coupled entity, rather than separable processes dominating the overt behavior.

RQA (Zbilut and Webber, 1992) is designed to detect subtle repetitive patterns in a trial series, used when data are noisy, irregular, and high dimensional (Zbilut et al., 2002; Pellecchia and Shockley, 2005; Marwan et al., 2007). It is based on procedures that visualize patterns of recurrence in a trial series, creating a matrix that shows recurrent aspects in the autocorrelation of the trial series. A variety of statistical measures are returned in an RQA, including the percentage determinism, entropy, trend, and the maximum line. *Percentage determinism* quantifies the degree of randomness of a process. High percentage of determinism implies that the future states of the system are determined by its previous and present states. *Entropy* represents the uncertainty based on Shannon's information entropy. This measure captures the degree of disorder that a system expresses. Systems made of components that operate independently, without any connection between them, should express a highly entropic behavior. The *maximum line* characterizes the system stability. Periodic signals produce long diagonal lines, chaotic signals generate very short diagonal lines, and stochastic signals cannot generate any diagonal line at all. Finally, the *trend* depicts the degree of the stationarity associated to the system. Values near to zero reflect stationarity, and values deviating from zero shows drift in the system (cf., Webber and Zbilut, 1994, 2005; Riley and Turvey, 2002; Turvey, 2007).

### Overview of experiments

Two tasks were used that involved hierarchical compound stimuli, a visual-search task and a visual-matching task. Both tasks have been used with children before, namely to investigate the importance of element sparcity in local/global processing (Kimchi et al., 2005). In Experiment 1 (visual search), children had to find the target among distractors, the target matching in local elements on some trials, and in global patterns on other trials. In Experiment 2 (visual matching), children had to decide whether a middle display matched with the right or the left answer option, with some trials featuring a match in local elements, and other trials featuring a match in global patterns. Adults were included as comparison group against which we can compare children's performance.

Both experiments had a large number of trials to mimic the duration of natural explorations during everyday tasks. The hierarchical compound items differed very little from trial to trial, to minimize distractions and allow for fast task performance. However, to avoid repetitiveness of trials, we manipulated the number of compound items per trial, as well as the number of elements per display (as was done in Kimchi et al., 2005). Outcome measures were accuracy and reaction time, to compare with previous results and to test for evidence of non-random coupling in the structure of the time series.

## Experiment I: visual search

Experiment 1 employed a search task in which participants had to search through a series of items and find the item that looked different from all the other ones. The target differed from the distractors either in local elements or in global shape. Filler trials had no target.

### Methods

#### Participants

Children were three girls and six boys between 6 and 9 years of age (*M* = 8.0 years, *SD* = 0.98), recruited from urban elementary schools serving Midwestern families from a wide range of socio-economic status. Adults were six women and three men between 18 and 36 years (*M* = 22.10 years, *SD* = 5.82 years), recruited from the introduction-to-psychology subject pool at a large Midwestern university. In return for participation, children received a small toy, and adults received partial course credit. All participants were native English speakers with no self-reported history of vision impairments.

#### Material

Elements were either black diamonds or black squares. Identical elements were arranged into a global pattern that formed either a diamond or a square (approximately 1.5 × 1.5 cm). Elements could be either large (5 × 5 mm) or small (2 × 2 mm), and global patterns consisted of four (2 × 2), nine (3 × 3), or sixteen (4 × 4) identical elements. Specifically, the 2 × 2 items always consisted of large elements, while the 3 × 3 and the 4 × 4 items always consisted of small elements.

**Figure 1:**
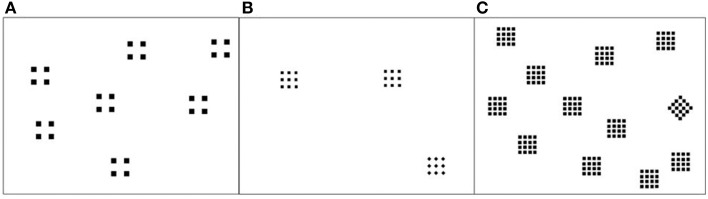
**Example trials in Experiment 1, showing different number of elements per item, and different numbers of items. (A)** target-absent trial. **(B)** element-different trial. **(C)** configuration-different trial.

On a given trial, 3–16 items appeared together, arranged randomly on a computer screen, with at least 1 cm distance between them. Figure 1 shows example trials with various numbers of items and different patterns. Distractors on a given trial were always square patterns composed of square elements. On element-different trials (Figure 1B), the target differed from the distractors only in the elements: the square pattern had diamond elements. In contrast, the target on configuration-different trials differed from the distractors only in global shape: the square elements were configured into a diamond configuration (Figure 1C).

There were 84 unique target-present trials, depending on the number of items per trial (3 to16), the number of elements in a pattern (4, 9, or 16) and the target type (configuration-different; element-different). Filler trials (Figure 1A) matched perfectly with the target-present trials, the only difference being that the target configurations was changed to match the distractor configurations. The resulting 168 trials made up one block of trials.

As warm-up, a series of black-and-white line drawings was used, including a fish, a monkey, and two flowers. Each drawing was about 1.5 × 1.5 cm, and they were arranged to create trials in which a target was present (e.g., a monkey was presented together with a series of fish). A numeric keypad was used to record participants' responses. The numbers 1 and 3 on the keypad were covered with the letters Y and N to represent the Yes and No options, respectively.

#### Procedure

The procedure in both experiments was approved by the local Institutional Review Board, and all steps of ethical treatment of human subjects were followed. Participants were tested individually, either in a quiet room at their school (children), or in the laboratory (adults). The experiment was carried out on either a 15.6″ Dell laptop (when testing took place at schools) or a 19″ flat-panel monitor Dell computer (when testing took place in the lab). Direct RT was used to present the stimuli and to record participants' responses and reaction time.

All instructions were presented on the computer, read by the experimenter. In order to familiarize participants with the search task, they were first presented with a series of trials for which the target (or the absence of a target) was pointed out explicitly. Participants were then presented with the numeric keypad and the two relevant keys. The instructions were: “Your task is to decide if the displays are different. Press Y for Yes. Press N for No.” Children were given four feedback trials (i.e., 2 element-different trials, one target-absent trial, and one configuration-different trial). In the case in which they pressed Yes (i.e., claiming that the displays are different), they also had to point to the display that was different. Adults were presented with only the last two of these feedback trials.

Prior to the experimental trials, participants were encouraged to “be as quick and precise as possible.” After approximately 10 testing trials, children were reminded to determine whether displays are different. Adults did not receive this reminder. Children were presented with four blocks of testing trials (672 trials total), and adults were presented with seven blocks (1176 trials). Trials within a block were presented randomly.

### Results and discussion

Two children and one adult did not complete the full set of trials (they responded to 613 (91.22%), 624 (92.86%), and 1159 (98.55%) trials, respectively). Given that they completed over 90% of the trials, their data was nevertheless included in the analyses. In a preliminary section, we will provide information about accuracy and reaction time, as a function of within-subject manipulations. While these analyses are not the focus of the paper, they are presented here nevertheless, as a means to comparisons to previous findings. We will then turn to the results obtained from the DFA and RQA.

#### Accuracy and reaction time

As was found before, children had lower accuracy than adults, both on target-present trials [*M* = 80 vs. 98 % correct; *F*_(1, 16)_ = 31.06, *p* < 0.01; η^2^_*p*_ = 0.66], and on filler trials [*M* = 88 vs. 99% correct; *F*_(1, 16)_ = 12.74, *p* < 0.01; η^2^_*p*_ = 0.44]. Similarly, children performed more slowly than adults, again on both the target-present trials [*M* = 1816 vs. 846 ms; *F*_(1, 16)_ = 42.42, *p* < 0.01; η^2^_*p*_ = 0.73] and on filler trials [*M* = 1927 vs. 1405 ms; *F*_(1, 16)_ = 9.96, *p* < 0.01; η^2^_*p*_ = 0.33].

**Figure 2:**
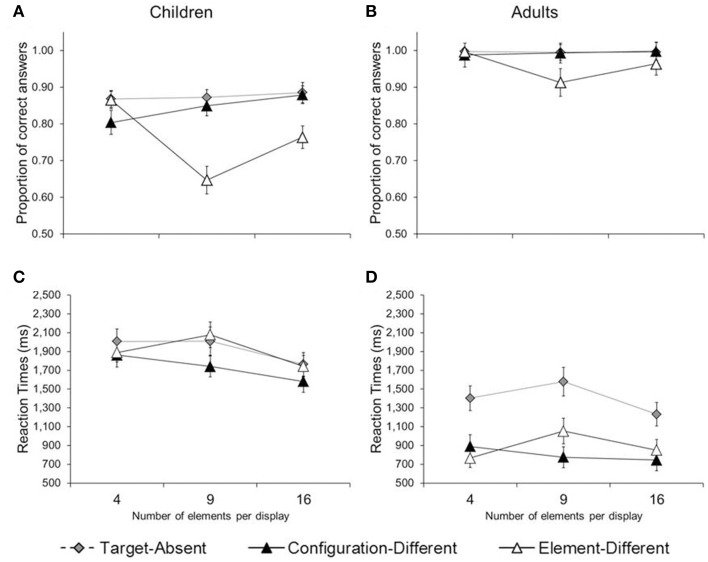
**Mean proportion of correct answers (A,B) and reaction times (C,D) in Experiment 1, separated by age group (children and adults), trial type (target-absent, configuration-different, and element-different), and number of elements per configuration (4, 9, and 16)**. Error bars illustrate the standard errors.

We were also able to replicate the interaction between number of elements and type of trial (configuration-different, element-different). Take children's accuracy, for example (Figure 2A, collapsed across the number of items per trial). An increase in number of elements (from 4 to 9 to 16) led to an increase in accuracy in configuration-different trials [*M*_4_ = 80; *M*_9_ = 85; *M*_16_ = 88; *F*_(2, 15)_ = 3.89, *p* < 0.04; η^2^_*p*_ = 0.34], while it led to a decrease in accuracy in element-different trials [*M*_4_ = 87; *M*_9_ = 65; *M*_16_ = 76; *F*_(2, 15)_ = 14.82, *p* < 0.01; η^2^_*p*_ = 0.66]. This double-dissociation between number of elements and trial type was also visible in adults (see Figure 2B), though far less pronounced given that adults performed largely at ceiling.

Reaction-time data too provided evidence for the double-dissociation. In children (Figure 2C), an increase in number of elements led to faster performance in configuration-different trials [*M*_4_ = 1862; *M*_9_ = 1741; *M*_16_ = 1582 ms, *F*_(2, 15)_ = 6.04, *p* < 0.02; η^2^_*p*_ = 0.45], while it led to slower performance in element-different trials, at least from 4 to 9 elements per item [*M*_4_ = 1888, *M*_9_ = 2078 ms, *p* < 0.01]. In adults (Figure 2D), an increase in number of elements led to faster performance in configuration-different trials [*M*_4_ = 889, *M*_9_ = 774, *M*_16_ = 744 ms, *F*_(2, 15)_ = 4.01, *p* < 0.04; η^2^_*p*_ = 0.35], while it led to slower performance in element-different trials, at least from 4 to 9 elements (*M*_4_ = 766; *M*_9_ = 1053 ms, *p*s < 0.01). Thus, children and adults were sensitive to the number of the elements of hierarchical patterns: they showed a local bias for few-element configurations and a global bias for many-element configurations.

To what extent does this sensitivity emerge from a coupling among many processes? To answer this question, we look at the outcome of the Detrended Fluctuation Analysis next.

#### Detrended fluctuation analysis (DFA)

A participant's reaction-time data (which included all of the trials, independently of type and accuracy) were submitted to DFA. For control purposes, we re-shuffled each time series, such that individual data points were re-ordered randomly. The reshuffling eliminates the sequential dependence of trials, and thus provides a baseline measure of coupling (i.e., chance structure). We then calculated the Hurst exponent for the original and the randomly reshuffled time series. The difference between these two Hurst exponents, for a particular participant, reflects the degree of coupling, over and above chance structure.

**Figure 3:**
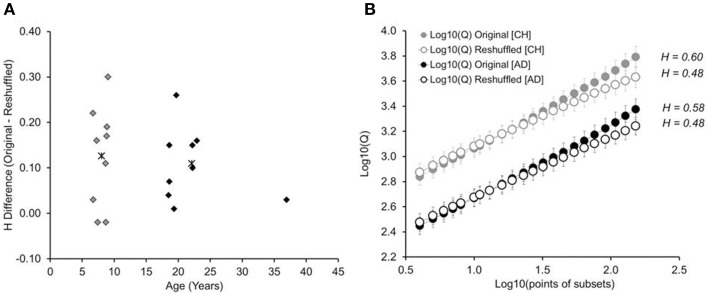
**(A)** Difference between the Hurst exponents determined for the original time series and the randomly reshuffled time series, shown as a function of participants' age. Asterisk show the average difference for children (gray) and adults (black), respectively. **(B)** Cumulative plots on log_10_-log_10_ coordinates, separated by age group (children, adults), and type of trial series (original and reshuffled). Error bars represent standard errors of average Qs. CH: children. AD: adults.

Figure 3A shows the difference in Hurst exponents (*H_originaltimeseries_*—*H_reshuffletimeseries_*), plotted against the age of the participant. Findings show similar distributions of *H* differences between children and adults, *p* > 0.69: While the *H* difference varied in size across children, the variability matched that found in adults. A 2-by-2 mixed-design ANOVA, with age group as the between-group factor, trial series (original, reshuffled) as the within-group factor, and *H* as the dependent variable, revealed an effect of trial series, *F*_(1, 16)_ = 26.81, *p* < 0.001; η^2^_*p*_ = 0.62: Hurst exponents were higher in the original trial series (*M* = 0.59, *SD* = 0.09) than the reshuffled time series (*M* = 0.48, *SD* = 0.04). This difference held up for each age group separately, *t*s > 3.14 (children: 0.60 vs. 0.48; adults: 0.59 vs. 0.48). There was no effect of age group or a significant interaction, *Fs* ≤ 0.20. The observed Hurst exponents were significantly higher than the Hurst exponent expected for of white noise (*H* = 0.50), both in children [*H* = 0.60, *t*_(8)_ = 2.89; *p* = 0.02] and adults [*H* = 0.59, *t*_(8)_ = 3.32; *p* = 0.01].

There is a caveat with determining the Hurst exponent for each participant individually. This is because such calculation depends on the presence of a linear relation on the log_10_-log_10_ plot, which was not given on the level of individual participants. Therefore, in order to test whether we can replicate the findings without relying on the log_10_-log_10_ plot of individual participants, we determined the cumulative log_10_-log_10_ plot for each age group, separated by original and reshuffled time series (see Figure 3B). The results mimic what was found with individual trial series: The cumulative Hurst exponent is higher for original than re-shuffled data series, with no difference between children and adults^1^.

#### Recurrence quantification analysis (RQA)

**Figure 4:**
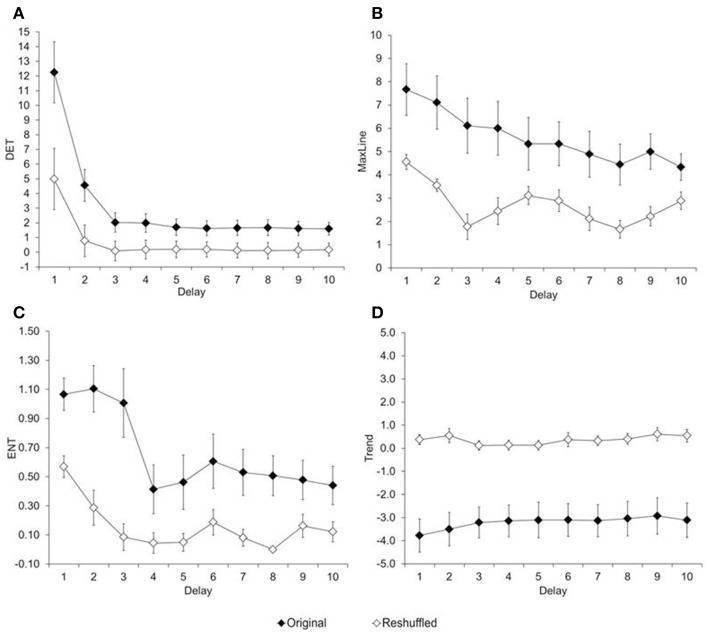
**Average of recurrence variables. (A)** Determinism, **(B)** Maximum Line, **(C)** Entropy, and **(D)** Trend estimated from original and reshuffle reaction times of children in Experiment 1.

To what extent is the variability in reaction time non-random? This question is particularly relevant for children's data, because their reaction-time variability appears random^2^. Recurrence variables were estimated with two embedded dimensions, a radius of 10, and plotted for lags between 1 and 10 trials. Figure 4 shows the resulting RQA measures (percent determinism, maximum line, entropy, and trend), plotted as a function of the chosen delay, and separated by type of trial series (original, reshuffled). Results show that each measure was higher for the original than the reshuffled time series, regardless of the delay, *t*s_(8)_ ≥2.35; *p*s ≤ 0.04.

## Experiment II: visual matching

To what extent do the results hold up in a different task? To address this question, we collected data for a second task, visual matching, where participants were asked to decide which of two answer options matched best with the middle display (see also Experiment 2 of Kimchi et al., 2005).

### Methods

#### Participants

Children were six girls and two boys between 6 and 9 years of age -year-olds (*M* = 8.24 years, *SD* = 1.07), and adults were seven women and one man (*M* = 22.11 years, *SD* = 6.79 years). Recruitment procedures were the same as in Experiment 1.

#### Materials

We again used elements of a particular shape (black squares or black circles), arranged into a pattern of a particular shape (square or diamond). There were 4, 9, or 16 elements per pattern (2 × 2, 3 × 3, and 4 × 4), and the sizes of elements and displays were comparable to what we used in Experiment 1.

**Figure 5:**
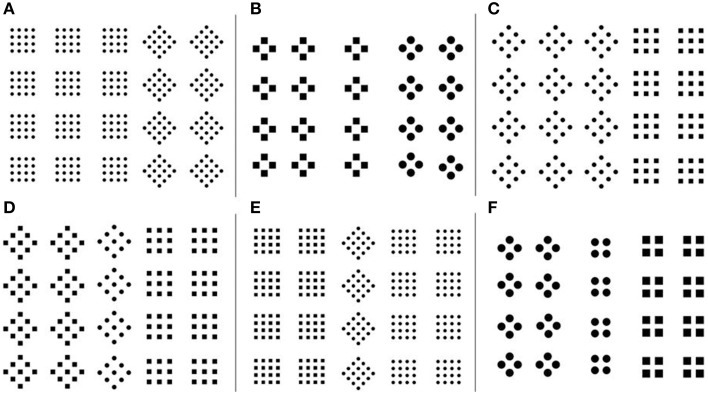
**Example trials used in Experiment 2. (A–C)** Perfect-match trials for which the answer options differed from each other in configuration only **(A)**, in element only **(B)**, or in both **(C)**; **(D)** Configuration-Match trial; **(E)** Element-Match trial; **(F)** Conflicting Trial.

Four identical items were arranged into a column, and five columns appeared on a given trial (see Figure 5 for example trials). The two left and the two right columns showed the answer options. In perfect-match trials, the middle column matched perfectly with one answer option, the two answer options differing from each other in global pattern only (Figure 5A), in element only (Figure 5B), or in both (Figure 5C). In configuration-match trials, the middle column matched with one answer option only in global patterns, the answer options differing from each other in configuration (Figure 5D). In element-match trials, the middle column matched with one answer option only in elements, the answer options differing from each other in elements (Figure 5E). Finally, in conflicting trials, the middle column matched with one answer option in global pattern, and with the other answer option in elements (Figure 5F). In this case, the two answer options differed in both configuration and elements.

Depending on trial type (3 types of perfect-match trials, 1 type of configuration-match trial, 1 type of element-match trials, and 1 type of conflicting trial), left-right arrangement, and the number of elements per configuration (4, 9, 16), the number of unique trials was as follows: 18 perfect-match trials, 6 element-match trials, 6 configuration-match trials, and 6 conflicting trials. Within a block, each trial was repeated four times, resulting in a total of 144 trials.

For familiarization, we used line drawings, geometrical shapes, and hierarchical patterns. Specifically, the line drawings showed animals (e.g., fish, monkey), plants (e.g., flower, fruit), and artifacts (e.g., airplane), scaled to approximately the same size. The geometrical shapes were triangles of different angles and orientations, and the configurations mimicked those of the experimental trials. A numeric keypad was used again, this time with letters L and R covering two keys, respectively. They represented the two answer options, left and right.

#### Procedure

The general set-up mimicked that of Experiment 1. During warm-up, participants were told: “In this game, you have to decide which side matches with the drawing in the middle. Look at the middle. Which side does it match with?” There were a series of warm-up trials to illustrate the task, illustrating, for example, that a “match” takes into account size and orientation. To prepare them for conflict trials (where there is no right or wrong answer), participants were told: “Sometimes it is very difficult to make a decision. Just make a guess.”

Testing started immediately. Before each trial, a fixation point appeared for 320 ms and reaction time and accuracy were recorded. Children were presented with four blocks (144 × 4 = 576 trials), and adults were presented with eight blocks (144 × 8 = 1152 trials). Trials within a block were presented randomly.

### Results

We will again present accuracy and reaction time first, after which we turn to the detrended fluctuation analysis and the recurrence quantification analysis.

#### Accuracy and reaction time

**Figure 6:**
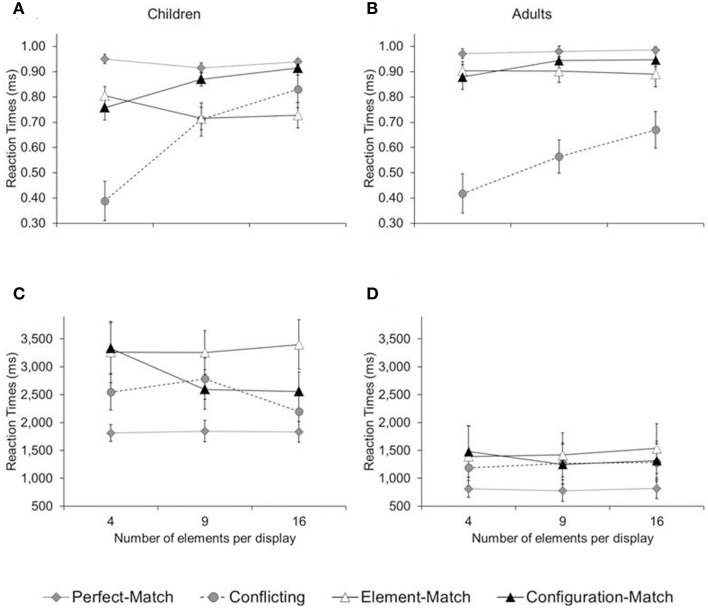
**Mean proportion of correct answers (A,B) and reaction time (C,D) in Experiment 2, separated by age group (children, adults), trial type (perfect-match, configuration-match, element-match, conflicting), and number of elements per configuration (4, 9, 16)**. Error bars illustrate the standard errors.

For scoring purposes of conflicting trials, we considered the correct choice to be the answer option that matched in configuration, rather than in element, with the middle column. Figure 6 depicts the resulting mean accuracy and reaction time, collapsed across number of items per trial.

Children were less accurate than adults, at least in the case of the perfect-match, element-match, and configuration-match trial [*M* = 84 vs. 93%; *F*_(1, 14)_ = 6.02, *p* < 0.02; η^2^_*p*_ = 0.30]. They were also slower than adults on these trials [*M* = 2655 vs. 1200 ms; *F*_(1, 14)_ = 10.14, *p* < 0.01; η^2^_*p*_ = 0.42]. For conflicting trials, children were slower than adults [*M* = 2509 vs. 1247 ms; *F*_(1, 14)_ = 7.29, *p* < 0.02; η^2^_*p*_ = 0.34], but there was no difference in accuracy [*M* = 64 vs. 55%; *F*_(1, 14)_ = 1.13, *p* = 0.31]. There was an interaction between trial type (configuration-match, element-match) and the number of elements per configuration—best visible in children's accuracy (Figure 6A): An increase in number of elements per configuration led to an increase in accuracy in configuration-match trials, [*M*_4_ = 76, *M*_9_ = 87, *M*_16_ = 91%; *F*_(2, 13)_ = 8.11, *p* < 0.01; η^2^_*p*_ = 0.55], but a marginal decrease in accuracy in element-match trials [*M*_4_ = 81, *M*_9_ = 72, *M*_16_ = 73% correct; *F*_(2, 13)_ = 3.13, *p* < 0.08; η^2^_*p*_ = 0.33]. This double-dissociation between number of elements and trial type was partially mimicked in adults, though far less pronounced given their tendency to perform at ceiling. There was an marginal increase in accuracy for configuration-match trials [*M*_4_ = 88, *M*_9_ = 94, *M*_16_ = 95% correct; *F*_(2, 13)_ = 3.24, *p* < 0.07; η^2^_*p*_ = 0.33], but no difference among element-match trials (*M*_4_ = 90, *M*_9_ = 90, *M*_16_ = 89 % correct; *p* > 0.78).

For conflicting trials, the number of elements per item had a substantial effect: the likelihood of choosing the global match increased steadily as the number of elements increased, both for children, [*M*_4_ = 39, *M*_9_ = 71, *M*_16_ = 83%; *F*_(2, 13)_ = 12.87, *p* < 0.01; η^2^_*p*_ = 0.66] and for adults, [*M*_4_ = 42, *M*_9_ = 57, *M*_16_ = 67%; *F*_(2, 13)_ = 4.82, *p* < 0.03; η^2^_*p*_ = 0.43]. For example, while children and adults performed at chance when there were only four elements per item, they consistently picked the answer option that matched in global pattern when there were 16 elements per item, *t*s_(7)_ ≥ 2.49, *p*s ≤ 0.05. Thus, both children and adults showed a local bias for few-element configurations and a global bias for many-element configurations.

The interaction between number of elements and trial type are further supported by reaction-time data, at least in children (see Figure 6C): While an increase in number of elements led to faster performance in configuration-match trials [*M*_4_ = 3332, *M*_9_ = 2593, *M*_16_ = 2556 ms, *F*_(2, 13)_ = 5.46, *p* < 0.02; η^2^_*p*_ = 0.46], the number of elements did not have an effect on element-match trials (*M*_4_ = 3264, *M*_9_ = 3258, *M*_16_ = 3398 ms, *p* > 0.52). There was no effect of element number on perfect-match trials, (*M*_4_ = 1812; *M*_9_ = 1846; *M*_16_ = 1834 ms, *p* > 0.93). In adults, performance was uniformly fast, with no interaction effect between number of elements and trial type, *F*_(2, 13)_ ≤ 0.82, *p*s ≥ 0.46.

#### Detrended fluctuation analysis

**Figure 7:**
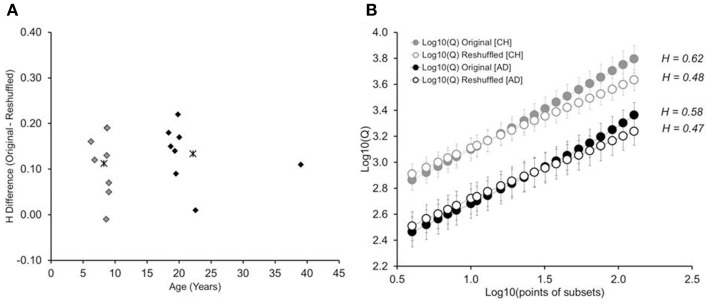
**(A)** Difference between the Hurst exponents determined for the original time series and the reshuffled time series, shown as a function of participants' age. Asterisks show the average difference for children and adults, respectively. **(B)** Cumulative plots of subset size against Q, in log_10_-log_10_ coordinates, separated by age group (children, adults), and type of trial series (original and reshuffled). Error bars represent standard errors of average Qs obtained for the group of eight participants in Experiment 2. CH: children and AD: adults.

We again submitted reaction-time data to DFA, as well as each participant's reshuffled time series. Figure 7A shows each participant's difference in Hurst exponents (*H_original_*—*H_reshuffled_*), plotted against the participant's age. Results mimic those obtained in Experiment 1: While there was some variability in the Hurst-exponent difference across children, children and adults had similar values, *p* > 0.54. The original trial series had higher Hurst exponents that their respective reshuffled trial series, both for children, *t*_(7)_ = 4.5, *p* < 0.01, and adults, *t*_(7)_ = 5.90, *p* < 0.01. Figure 7B shows the log_10_-log_10_ cumulative plot, determined across participants. Again, we found higher cumulative Hurst exponents for the original than reshuffled time series. And the observed exponents were significantly higher than white-noise Hurst (*H* = 0.50), again in both children [*H* = 0.59, *t*_(7)_ = 3.51; *p* = 0.01], and in adults [*H* = 0.62, *t*_(7)_ = 4.23; *p* = 0.004].

#### Recurrence quantification analysis

**Figure 8:**
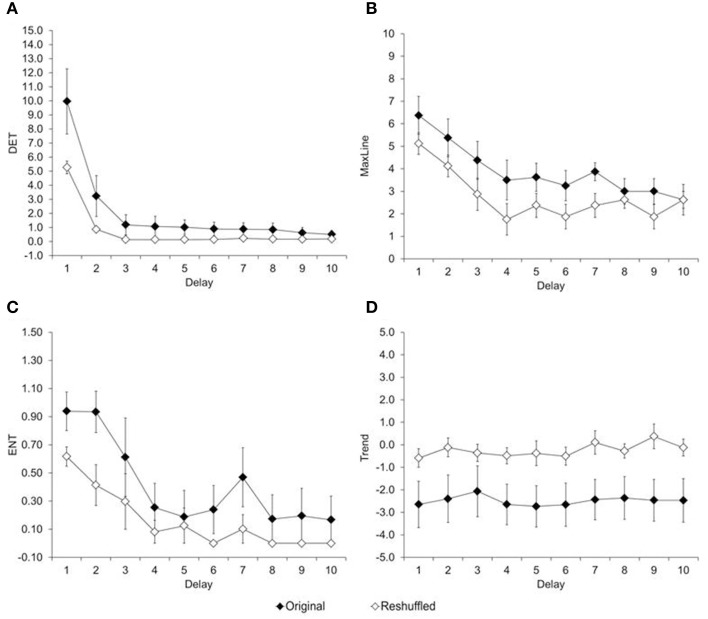
**Average of recurrence variables. (A)** Determinism, **(B)** Maximum Line, **(C)** Entropy, and **(D)** Trend estimated from original and reshuffle reaction times of children in Experiment 2.

Recurrence variables were estimated with two embedded dimensions, a radius of ten, and plotted between 1 and 16 trials lags (Figure 8). A repeated-measure ANOVA shows that recurrence variables decrease quickly in the extents that delay between trials increase, *F*s_(6, 180)_ = 47.66, *p* < 0.01, η^2^_*p*_ ≥ 0.61. However, regardless of the delay, the original time series had higher determinism (Figure 8A); stability (Figure 8B); entropy (Figure 8C) and non-stationarity (Figure 8D) than their respective reshuffled time series, *t*s_(7)_ ≥ 2.67, *p*s ≤ 0.06. Like in Experiment 1, the degree of entropy associated to the system was in the range in which complexity can emerge. Similarly, the system stability was in the range of periodic to chaotic signals, different from what would be indicative of a stochastic system. Finally, the degree of stationarity was low, indicative of a system that is drifting, rather than be stationary.

## General discussion

Two tasks were used to investigate how children and adults perceive hierarchical compound items, items for which elements give rise to a higher-order Gestalt. There was no explicit instruction about whether to focus on individual elements or on the overall Gestalt, mimicking perception in every-day tasks. Trials differed in whether the items were varying in their elements or their overall Gestalt, bringing about a perceptual mode that takes into account all levels of order. The question was whether this perceptual mode has the signature of adaptive coupling of multiple processes (Kello and Van Orden, 2009; Wallot et al., 2015).

Results provide positive evidence for this question. Even though there were notable differences between children and adults, in both accuracy and reaction time, the analyses revealed striking similarities across age groups. For both children and adults, the DFA yielded Hurst exponents that were significantly above the exponents obtained for the reshuffled time series (and significantly above randomness). Importantly, the distribution of exponents did not differ between children and adults. Both children and adults showed a fluidity of performance that is characteristic of self-organizing cognitive systems (Wijnants et al., 2009).

Additional evidence for a coupled process of local/global perception comes from the RQA conducted with children's reaction-time data. All measures pointed in the same direction: There was a clear difference between the original trial series and the reshuffled time series, showing that variability in reaction time, from one trial to the next, was far from random. Time series showed a configuration resistant to a growing number of delays that could not be explained by the presence of independent processes.

Our findings are relevant in several ways: (1) they replicate previous results with adults, (2) they demonstrated long range correlations and patterns of recurrence in children's response time during local/global perception, and (3) they add to the conversation about whether local/global perception is sub-served by separable processes. Each of these aspects in discussed in turn.

### Perception of hierarchical compound items in adults

Our previous research established that the perception of hierarchical displays has the signature of self-organized adaptability (Castillo et al., 2015): Alpha exponents returned by a spectral analysis were above what would be expected by chance, even in the most difficult task version (e.g., when elements were unfamiliar and trial order was unpredictable). The current results extend those findings, namely by using two new tasks and subjecting the data to DFA.

The main difference between the previous and the current kinds of task was in the instructions: In previous tasks, adults were explicitly asked to focus on a specific level in the hierarchy of orders. For example, they were asked to decide whether two items shared an individual element. In contrast, in the current tasks, no explicit instructions were given about what level to focus on. Instead, participants were merely asked to compare items. Their high accuracy across all types of trials, whether the match was in local element or global Gestalt, implies that adults paid attention to both levels of order. Thus, we succeeded in bringing about a kind of perception that required a switch between local and global aspects of the displays. And indeed, this kind of perception had the signature of adaptive interdependence of different processes.

Across the two different experiments of the current study, the size of the Hurst exponents corresponds to the range of alpha exponent found in Castillo et al. (2015). They show compatibility of fractal measures, at least when it comes to spectral analyses and detrended fluctuation analyses, in line with what was found before (e.g., Kiefer et al., 2009). Together, these findings provide further evidence that skilled perception of visual scenes is guided by the self-organization of coupled processes (cf., Aks and Sprott, 2003; McIlhagga, 2008; Stephen and Mirman, 2010; Coey et al., 2012). They suggest that integration and segmentation are part of an ongoing conglomerate of many processes: A kind of emergent coupling that cannot be described as separable components (Wallot et al., 2015). To what extent does the same apply for children?

### Development of local/global processing

In terms of development, the main finding was that children and adults had similar Hurst exponents, across both the search task and the matching task. Thus, whether children were asked to search for the display that looks different, or match a set of displays to one group or another, the magnitude of Hurst exponents stayed stable across age. One could argue that our design had too little power to detect a developmental difference. However, such claim would run counter to our findings with accuracy and reaction time: Here we found clear developmental changes across age. It appears instead that children demonstrate a level of coordination that is similar to that of adults. This conclusion is further supported by the results returned by the recurrence quantification analysis, showing moderate levels of determinism, system stability, entropy and non-stationarity.

Previous studies that investigated developmental changes in Hurst exponents found a moderate increase with age, whether the task was to tap the index finger at a specific frequency (Kiefer et al., 2009) or to advance a text while reading (Wallot et al., 2014). Those results were interpreted as evidence for an increase adaptive coupling of relevant processes, perhaps due to an optimization of constraints that allows for a more stable coordination (Scherf et al., 2009). In contrast, the visual perception of hierarchical compound items investigated in the current study does not show the same level of improvement. It is possible that the age range chosen for the current studies is too narrow to detect developmental differences. It is also possible, however, that even younger children demonstrate an adaptive self-organizing process of local/global perception, despite lower levels of accuracy and slower performance.

### Is the idea of separate processes of integration and differentiation justified?

Finding non-random Hurst exponents, in the time series of local/global performance; hints at the presence of a system that cannot be decomposed into its constituents (Kelso, 1995). Thus, our findings add to the growing dissatisfaction with postulating different processes to explain different behavior (Aks et al., 2002; Aks and Sprott, 2003; Van Orden et al., 2003; Stephen and Mirman, 2010; Coey et al., 2012). Building on these arguments, we suggest that the fault line in local/global processing runs between easy and difficult tasks, as explained next.

It is easy to focus on an overall pattern when the elements are very small (e.g., face perception), and it is easy to ignore an overall pattern when elements are large and salient (e.g., super-ordinate categorization) (Walton, 2014). Vice versa, it is difficult to ignore large elements to detect the overall pattern (e.g., abstract reasoning), and it is difficult to focus on small element while ignoring the larger order (e.g., perceptual learning) (Goldstone, 1998). Indeed, the developmental trajectory of the easy task is flat, while the trajectory of the difficult task is steep (Kimchi, 1990, 1998). This difference in trajectory is the result of differences in constraints on the entire system, rather than in different underlying processes. In the easy task, there are multiple mutually enforcing constraints available to guide performance. In a difficult task, in contrast, performance depends on supplying the necessary constraints, in the form of top-down control, to support performance (Evans, 2003).

Our evidence of a self-similar fractal system in local/global processing suggests that we need to take into account a multitude of processes, not just those of integration and differentiation, to explain fluid perception in hierarchical scenes. And the systematic control of these processes is an emergent property sustained by multiplicity feedback mechanisms, phenomenon usually conceptualized like allometric control (West and Griffin, 1999; West, 2010).

In sum, we found support that performance in the local/global task is the result of an emergent and self-organized coupling of a multitude of processes in the task-actor system, captured by the fractal scaling exponent and recurrence variables of reaction time data. Integration and differentiation are both required to make sense of the surrounding, coherently coupled to provide adaptive fluidity in visual perception. Developmentally, it is likely that this self-organization is most clearly visible in a task that requires little top-down control. It might show a protracted development in tasks that require more deliberate control in a context with only few supportive constraints.

### Conflict of interest statement

The authors declare that the research was conducted in the absence of any commercial or financial relationships that could be construed as a potential conflict of interest.

## Footnotes

^1^The cumulative plot looks different for adults vs. children, due to the difference in total number of trials.

^2^Adults are not included in this analysis because their recurrent measures estimated from observed trials decreased to the same low level of reshuffled trials with delays equal or more than 2.
